# Supplementary material for: Biomarker Genes Discovery of Alzheimer’s Disease by Multi-Omics-Based Gene Regulatory Network Construction of Microglia
Source: Brain Sci. 2022 Sep 5;12(9):1196. doi: 10.3390/brainsci12091196 (PMC9496783; doi:10.3390/brainsci12091196)
Supplement: Supplementary file 1 [file brainsci-12-01196-s001.zip › brainsci-1844124-Supplementary File S1.pdf]

Since the age of patients over 90 years was shown in the clinical data, we set the starting point of observation as the time of diagnosis for each patient, and the end point of observation as 90 years.

The survival status of patients with age at death above 90 years was alive, while the survival status of patients with age at death below 90 years was dead, resulting in survival times for 82 patients.

Clinical data downloaded from the AMP-AD database were sorted and screened, and then survival times and clinical characteristics were obtained for 82 patients (braaksc, ceradsc, dcfdx_lv: summary of clinical cognitive diagnosis at last visit).
